# Effective health promoting school for better health of children and adolescents: indicators for success

**DOI:** 10.1186/s12889-019-7425-6

**Published:** 2019-08-13

**Authors:** Albert Lee, Amelia Siu Chee Lo, Mei Wan Keung, Chi Ming Amy Kwong, Kwok Keung Wong

**Affiliations:** Centre for Health Education and Health Promotion, The Chinese University of Hong Kong, 4th Floor, Lek Yuen Health Centre, 9 Lek Yuen Street, Shatin, New Territories Hong Kong

**Keywords:** Health promoting school, Indicators, Evaluation, Adolescent and children

## Abstract

**Background:**

Improvement of health literacy, health behavioural change, creating a supportive physical and social environment to be more conducive to health should be the focus of child and adolescent public health. The concept of Health Promoting School initiated by World Health Organization aims to move beyond individual behavioural change and to consider organisational structure change such as improvement of the school’s physical and social environment. The aim of this study is identification of the key indicators for successful implementation of Health Promoting School by analysing the findings of the school health profile based on the structured framework of Hong Kong Healthy School Award Scheme and the health status of students investigated by the Hong Kong Student Health Survey.

**Methods:**

This is a retrospective correlation study. Analysis of variance (ANOVA) was utilised to analyse for significant improvement of school health profile measured at baseline (*n* = 104) and among those schools implemented the Hong Kong Healthy School Award Scheme (*n* = 54). Those indicators showing statistical significance were chosen to be part of the core indicators reflecting effective Health Promoting School. Each of those selected core indicators was then correlated with the related student health outcomes measured by the Hong Kong Student Health Survey Questionnaire to further identify the core indicators.

**Results:**

A total of 20 core indicators among all the six Key Areas of Health Promoting School (6 indicators under action competencies, 2 under community link, 2 under physical environment, 2 under social environment, 4 under healthy school policies, 1 under services of school health protection) have been identified with the method mentioned above.

**Conclusions:**

This study has identified the indicators with most significant impact on a wide range of health related outcomes. Those are key indicators for motivating positive change of the schools and students. They can be considered as school performance indicators to help schools embarking their Health Promoting School journey as another key education objective.

**Electronic supplementary material:**

The online version of this article (10.1186/s12889-019-7425-6) contains supplementary material, which is available to authorized users.

## Background

In 2011, the United Nation adopted ‘The Declaration of the High-level Meeting of the General Assembly on the Prevention and Control of Non-communicable Diseases (NCD)’, and actions to reduce risk factors and creating health promoting environments became the focus of this agenda [1]. Behavioural, environmental, occupational, and metabolic risks factors such as high cholesterol, high blood pressure, obesity, smoking and alcohol, can explain half of global mortality and more than one-third of global DALYs providing many opportunities for prevention especially at early stage of life [2].

However, we are still observing high prevalence of those youth health risk behaviours in both developed and developing countries with around 30 to 40% of young people who had drunk alcohol and around 30 to 50% taken drug in Australia and US, and around 80% of secondary school students have been found to be physically active in Hong Kong, Macao, Taipei and US [3–5].

### Rationale for Core indicators of health promoting school

The concept of Health Promoting School (HPS) initiated by WHO aims to move beyond individual behavioural change and consider organisational and structural change such as improving the school’s physical and social environment, its curricula, teaching and learning methods [6, 7]. The WHO HPS framework is only an outline. Langford et al. (2014) conducted Cochrane Review of WHO HPS framework for improving health and well-being of students and their academic achievement based only on 67 included cluster-randomised controlled trials (RCTs) taken place at the level of school, district or other geographical area [8]. The RCT design does not necessary lend itself to outcomes involving organizational or structural change as the statistical assumptions underpinning RCT are not valid to reflect organisational or structural change so limited conclusion could be drawn [9]. Would HPS initiatives lead to immediate change at individual level? One would argue that potential markers of success are associated with the process [10]. Therefore, indicators of HPS should highlight the ways in which schools would be able to adopt HPS principles successfully and the conditions to be in place to flourish [10]. A boarder perspective on evidence is needed in dealing with the complexity of school system [11, 12]. The English Wessex Healthy School Award Scheme (WHSA) [13] and the Hong Kong Healthy School Awards Scheme (HKHSA) [14] have developed detailed system to analyse whether each individual school has reached the standard to be a model HPS, reflecting a more holistic appreciation and understanding of all the effects of school-based health promotion. Both schemes have shown positive award-related changes [15, 16].

Patton et al. (2010) called for global agenda of adolescent health to include data beyond mortality to include information about adolescent health in the social, cultural, and economic contexts in which young people grow [17]. A study on health-related Sustainable Development Goal (SDG) targets has highlighted the need for boarder public health programmes and policies on behavioural risk factors with multi-sectoral commitments and investments [18]. An ecological model of health promotion is needed to address the complex interaction of environmental, organisational, and personal factors such as healthy setting approach, recognising the contextual issue as well as investment in social systems in which people spend their daily lives. [19, 20]. HPS framework is enabling school to become an appropriate setting for health promotion so it needs a clearer translation for action to create a new era for school health [21]. The schemes of WHSA and HKHSA have provided the structured framework for development as well as system of monitoring and evaluation, and also recognition of achievements [22, 23]. Some core indicators are needed for a wider implementation of HPS especially in developing countries as starting point because not many of their schools are able to implement HPS in its entirety [24, 25]. Paper by Joyce et al. (2017) asserts the importance of data monitoring, such as audits adopted in HKHSA to motivate changes [25]. Hong Kong has scaled up and sustained the HPS movement over the last two decades to enable study to be conducted in identification of the key indicators for successful HPS practice based on the data collected over a period of time.

## Methods

### Data source and study instruments: school level data

The structured framework of HKHSA is used for evaluation of efficacy incorporating the four types of indicators, inputs, process, impact and outcomes suggested by Nutbeam [26] to measure the success of health promotion (Table 1) [14, 23]. It reflects a more holistic appreciation and understanding of all the effects of school based health promotion [27]. The HKHSA covers six key areas (healthy school policies, school’s physical environment, school’s social environment, community links, action competencies for healthy living, and school health care and promotion services) suggested by WHO Guidelines [6, 14, 28]. The indicators of student health profile describe the health status of students including life satisfaction and emotional health, and health behaviours. The school health profile describes the school environment (physical and social), healthy policies, pedagogy on health training, and organisational practices. One would then analyse the indicators reflecting school health profile correlating with better health related outcomes based on student health profile.

**Table 1:** Indicators and measuring instruments for the different types of outcomes for health promotion [14, 23]

Types of outcomes	Indicators to be measured	Measuring instrument
Health and social outcomes	Depressive symptoms, life satisfaction, perceived health status, perceived academic achievement	Validated questionnaires: Satisfaction with Life Scale (LIFE), Depression Self-Rating Scale (DSRS), Youth Risk Behaviour Survey (YRBS).
Intermediate outcomes	Attitudes, lifestyles and risk behaviours School environment and school ethos School health services	Questionnaires to students and schools, school observation, documentary review, interviews
Health promotion outcomes	Health skills and knowledge, and self-efficacy School health policies Networking with parents, the local community and other schools to launch health programmes	Questionnaires to students and schools, curriculum review, documentary review, individual or focus group interviews, participant observation
Health promotion actions	School timetable for health education activities (formal and extra-curricular) PTA and community involvement	Documentary review

A number of components under each key areas and the respective set of indicators with contextualisation specific to Asian Pacific countries (Additional file 1: Appendix 1) were developed with guidelines based on extensive local and oversea literature and documentary reviews [14, 16, 28]. Points were given for each indicator to calculate an overall score for each component under each respective key area as well as the overall score for each key area. The overall score for each component and each key area was calculated as percentage reflecting the level of achievement under each respective component and key area to build up a school health profile [29].

There is a strong body of evidence and theoretical framework in identifying the indicators that are relevant, adaptable and achievable reflecting school-based initiatives as well as involvement of parents, school managers and community, and also teacher training according to past and recent international practices [12–14, 30–34]. The system of accreditation of HKHSA had undergone process of validation by:

- Face validation: Pilot testing was done with principals and teachers from 18 primary and 19 secondary schools who had basic understanding of HPS [23]
- Content validation: Local and international HPS experts (6 international experts) provided valuable comments on the contents [23, 35]. There were two individual expertise teams: one was combined with veteran members (second and third authors of reference 35) who had been involved in the development of the HPS Performance Indicators, and was responsible for content validation. The other team was combined with the personal experience of the experts in Healthy School Award schemes, who were responsible for observing the process of accreditation and benchmarking.
- Criterion validation: HPS experts experienced in Healthy School Award schemes with publications on HPS evaluation [12, 32], examined the process of accreditation and benchmarking with international standards taken into account of the local context [23]

Construct validation was conducted by analysis of the correlation of school performance in and between elements, components and the HPS key areas, and overall performance, measured by the different levels of indicators, among schools that implemented the HKHSA. Positive correlation between the element and the component level performance, the component and the key area performance, and the overall school performance in HPS was observed and published [23].

The schools were categorised into different levels of achievement (gold, silver and bronze award) based on their school profiles, and analysed the areas of strength and weakness according to the different components of guidelines of HKHSA [29, 36].

### Student surveys

A system of surveillance of student health status based on the Hong Kong Student Health Survey Questionnaire (HKSHQ) incorporating the US Centre for Disease Control and Prevention (CDC) Youth Risk Behavioural Surveillance [3, 37–39] and WHSA [13] adapted by Centre for Health Education and Health Promotion of the Chinese University of Hong Kong (CHEP) [35, 39] with continuous refinement as a tool for assessing student health status/health related outcomes [40, 41]. K6 scale by Kessler and colleagues [42] has been used to assess emotional disturbance [40] as well as Life Satisfaction scale by Huebner and colleagues [42] as part of HKSHQ building up the student health profile. Primary 4 (aged 9 to 10 years) and Secondary 3 (aged 14 to 15 years) students were recruited for the survey in each primary and secondary school respectively.

### Data collection at school level

In 2010, the Quality Education Fund commissioned CHEP to establish the Quality Education Fund Thematic Network of Healthy School (QTN Healthy School) aiming to establish a ***school network*** for sustaining the Healthy School movement territory wide. Making use of the school health profile developed by CHEP as described in previous section [29, 33, 34], the performance of schools achieving HPS standard was assessed by their performance among the six key areas at baseline and re-evaluated again for Healthy School Award (Award). Data were collected on performance of different components of six key areas among the 104 schools when they first joined the network as baseline assessment and the performance of those schools with Award (54 schools).

### Data collection at student level

Primary four students and secondary three students were selected among those schools to analyse the changes in student health behaviours, self-reported health status and emotional health for consistency and to understand the cumulative impact of HPS at this mid-point of schooling making use of HKSHQ [35, 40, 41]. A previous study had shown improvement of student health, and also the school culture and organisation after 1 year [39]. Activities taken place in schools during the interim period included student leadership training, teachers training on HPS, and sharing of HPS good practice among teachers provided by CHEP.

Survey and Behaviour Research Committee of the Chinese University of Hong Kong approved the QTN Healthy School Study on tracing student health on 28 March 2011.

### Data analysis at school level

T-test statistics was utilised to analyse for significant improvement of school health profile measured at baseline and among those schools with Award with level of statistical significance at 0.05. The full performance profile consists of over 90 indicators, and among those, there are still many indicators with significant change in mean scores measured at baseline and at time of award. Therefore, those indicators with significant mean score difference more than 0.25 were chosen to be part of the core indicators reflecting effective HPS as they would represent certain unique features of achievement in accomplishing overall healthy school environment.

The creation of school health profile enables categorisation of schools into different levels of achievement using the cut-off for different levels of Award, gold, silver and bronze [34]. The difference of scores among schools with different levels of Award for each component of respective key area was then analysed by ANOVA with level of statistical significance at 0.05. Those indicators with statistical significance were also chosen to be part of the core indicators.

### Data analysis of correlation between indicators of school health profile and health related outcomes of students

In order to streamline the core indicators, each of those selected core indicators was correlated with related student health status measured by HKSHQ, i.e., indicators reflecting school social environment were correlated with emotional health and life satisfaction, indicators reflecting healthy eating policy and healthy eating environment were correlated with eating behaviours. The correlation was analysed by Pearson correlation coefficient, and analysis of primary and secondary schools was conducted separately.

## Results

As described in data analysis section, the basic requirements for HPS were identified by t-test are illustrated in Table 2, i.e., those elements/indicators showing significant mean score difference more than 0.25. Under the key area, School’s Physical Environment, the indicator ‘School has a system in place to ensure that all food sold or served in school promote healthy eating (PE 4.1)’ was also chosen even though the difference of mean between Baseline and Award was found to be less than 0.25. No indicators under school physical environment were found with the difference of at least 0.25 and only PE 4.1 was found to have the largest difference between Baseline and Award.

**Table 2:** Basic Requirements for HPS identified by t-test

Element	Mean ± S.E. in Baseline *n* = 104	Mean ± S.E. in Award *n* = 54	Mean score difference
Healthy School Policies			
1.4 Related personnel were consulted in the drawing up, implementing and monitoring the school health policies	0.34 ± 0.01	0.61 ± 0.02	↑0.27
School’s Physical Environment			
4.1 School has a system in place to ensure that all food sold or served in school promote healthy eating	0.51 ± 0.02	0.69 ± 0.02	↑0.18
School’s Social Environment			
3.2 School has a system in place to look after students and staff with emotional needs and/or unexpected traumatic life events	0.45 ± 0.02	0.74 ± 0.02	↑0.29
Action Competencies for Healthy Living			
4.3 School provides health-related information and resources for family members and the community	0.40 ± 0.02	0.78 ± 0.03	↑0.37
2.1 School uses a variety of innovative and student-orientated strategies and formats when implementing health education and promotion activities	0.48 ± 0.02	0.84 ± 0.02	↑0.35
1.1 School adopts a systematic approach to conduct health education	0.60 ± 0.01	0.88 ± 0.01	↑0.28
Community Links			
3.4 School supports staff to participate in various exchange activities in health education	0.25 ± 0.03	0.63 ± 0.03	↑0.38
3.2 School links with community bodies and works with them to promote community health education activities	0.51 ± 0.02	0.85 ± 0.02	↑0.34
1.2 School consults parents for recommendations on Healthy School development & encourages their active participation in the joint discussion on the formulation and review of Healthy School policies	0.26 ± 0.01	0.53 ± 0.02	↑0.27
School Health Care and Promotion Services			
6.1 School actively promotes occupational health and support related training	0.50 ± 0.02	0.79 ± 0.03	↑0.28
1.2 School encourages students to be immunised against appropriate infectious disease and their immunization status should be properly documented and followed up.	0.40 ± 0.03	0.64 ± 0.03	↑0.25
2.1 School encourages students to have health screening at least once a year with a monitoring system in place	0.62 ± 0.03	0.82 ± 0.03	↑0.20

*p*-value < 0.05

After identification of the basic requirements for HPS by t-test, ANOVA was conducted to analyse the difference of scores among schools with different levels of Award for each indicator of respective key area with level of statistical significance at 0.05. Table 3 provides the summary results of those indicators under different key areas showing statistical significance at level of 0.05 among schools with different level of awards by different level of analysis by ANOVA (please refer to Additional file 2: Appendix 2 for results of analysis). Correlation of those indicators with significant improvement as shown in Tables 2 and 3 was analysed for correlation with health-related outcome of students. Additional file 3: Appendix 3 provides all the results of correlation between those selected indicators under each key area with health-related outcomes. Table 4 shows results of correlation analysis of those selected indicators under each key area with health-related outcomes. Table 5 lists the key indicators for motivating change under each respective key area showing significant impact on health behaviours.

**Table 3:** The elements/indicators under different key areas showing statistical significance at level of 0.05 among schools with different levels of Award analysed by ANOVA

Element	
Healthy School Policies	
2.1 Healthy Eating	
2.2 Safe School	
2.3 Harmonious School	
2.4 Active School	
School’s Physical Environment	
1.1 School ensures students’ safety whenever students are under their care	
1.4 School has a system in place for the management of emergencies and natural disasters and ensure that all relevant personnel being informed	
1.5 School ensures fire safety	
1.7 School ensure a safe and healthy workplace for staff	
School’s Social Environment	
2.2 School has a system for the prevention, and management of unacceptable behaviour in school both among students and encourages staff to set personal examples for cultivating students’ positive actions	
Action Competencies for Healthy Living	
1.3 School tries to ensure all students have opportunities to actively engage with each topic, according to their age	
3.2 There are school staff who received professional training in health education or participated discussions on the development of health promoting school	
3.3 School staff participate in different health education workshops or seminars, and have opportunities to collaborate with other teachers and exchange ideas to enhance the teaching of health	
3.4 School provides diversified health education resources for staff, and such resources are well organised and managed	
Community Links	
2.2 School consults community members or groups that possess substantial understanding of the school for recommendations and/or professional advice on Healthy School development and involves them in assessing school’s developmental needs and/or discussing arrangements for corresponding plans and projects	
School Health Care and Promotion Services	
2.3 There was a provision of basic health care services and management	

*p*-value < 0.05

**Table 4:** Pearson correlation coefficients between student health related outcomes using measures by Hong Kong Student Health Survey Questionnaire (HKSHQ) and core indicators of the six key areas (** *p*-value < 0.01, * *p*-value< 0.05)

(a) Action Competencies (AC) for Healthy Living and Health Related Outcomes								
HKSHQ measures	Primary schools						Secondary Schools	
	AC1.1^1^	AC1.3	AC2.1	AC3.2	AC3.3	AC4.3	AC1.1	AC2.1
% of students think they are having good academic performance in past 12 months	0.175	0.138	0.085	0.155	0.260*	0.023	0.367**	0.121
% of students think they are having good health status over past 30 days	0.314**	0.349**	0.234*	0.256*	0.276**	0.118	0.108	0.003
% of students that often obey traffic signals	0.258*	0.034	0.229*	0.075	0.259	0.248*	0.400**	0.308*
% of students that often put on seatbelts	0.166	0.004	0.126	0.171	0.230*	0.171	0.370**	0.275*
% of students having enough vegetable every day	0.152	0.186	−0.011	0.236*	0.253*	0.001	−0.055	−0.242
% of students having enough fruit every day	0.243*	0.303**	0.099	0.264*	0.269*	0.158	0.258	0.147
% of students having crisps more than 4 times per week	−0.149	−0.348**	−0.069	− 0.136	−0.029	− 0.046	−0.267*	0.058
% of students having candies more than 4 times per week	−0.266*	−0.487**	− 0.177	−0.167	− 0.179	−0.075	− 0.427**	−0.110
% of students having soft drink more than 4 times per week	−0.326**	−0.356**	− 0.192	−0.287**	− 0.166	−0.214*	− 0.193	−0.158
% of students having preserved meat more than 4 times per week	−0.211*	−0.291**	− 0.101	−0.295**	− 0.131	−0.064	− 0.395**	−0.137
% of students having enough physical activity	0.330**	0.281**	0.206	0.240*	0.280**	0.318**	0.094	−0.032
Mean K6 score of students	−0.323**	− 0.359**	−0.205	− 0.362**	−0.277**	− 0.246*	−0.372**	− 0.307*
% of students smoke	−0.078	0.080	−0.046	− 0.248*	−0.070	− 0.057	−0.115	− 0.267*
Mean Life satisfaction score of students - family life	0.200	0.125	0.271*	0.236*	0.271*	0.299*	0.062	0.245
Mean Life satisfaction score of students - friendship	0.066	−0.035	0.098	0.231	0.202	0.237*	−0.068	0.231
Mean Life satisfaction score of students - themselves	0.262*	0.039	0.348**	0.061	0.376**	0.459**	0.126	0.220
Mean Life satisfaction score of students - living environment	0.218	0.042	0.225	0.285*	0.230	0.338**	0.139	0.271
Mean Life satisfaction score of students - overall life	0.155	0.034	0.193	0.186	0.259*	0.347**	0.107	0.291*
(b) Community Links (CL) and Health Related Outcomes								
HKSHQ measures	Primary schools		Secondary schools					
	CL1.2	CL2.2	CL2.2					
% of students think they are having good health status over past 30 days	0.110	0.370**	0.274*					
Mean Life satisfaction score of students - family life	0.268*	0.328**	0.040					
Mean Life satisfaction score of students - themselves	0.235*	0.305**	0.280					
Mean Life satisfaction score of students - living environment	0.193	0.255*	0.133					
Mean Life satisfaction score of students - overall life	0.155	0.292*	0.128					
(c) School’s Social Environment (SE) and Health Related Outcomes								
HKSHQ measures	Primary schools		Secondary Schools					
	SE2.2	SE3.2	SE3.2					
% of students think they are having good academic performance in past 12 months	0.223*	0.037	0.174					
% of students having enough physical activity	0.252*	0.190	0.006					
% of students having K6 score > 12 (indicating poor mental health)	−0.259*	−0.241*	−0.300*					
Mean K6 score of students	−0.211*	−0.291**	− 0.248					
(d) School’s Physical Environment (PE) and Health Related Outcomes								
HKSHQ measures	Primary Schools		Secondary schools					
	PE1.1	PE4.1	PE1.1	PE4.1				
% of students having enough fruit every day	0.119	0.164	0.194	0.345**				
% of students having soft drink more than 4 times per week	−0.278**	− 0.282**	−0.046	0.082				
% of students having enough physical activity	0.216*	0.239*	0.248	0.055				
Mean K6 score of students	−0.227*	− 0.183	−0.284*	− 0.302*				
Mean Life satisfaction score of students - living environment	0.265*	0.220	0.335*	0.072				
Mean Life satisfaction score of students - overall life	0.258*	0.183	0.196	0.110				
(e) School Health Care and Promotion Services (HS) and Health Related Outcomes								
HKSHQ measures	Primary Schools	Secondary Schools						
	HS2.3	HS2.3						
% of students think they are having good health status over past 30 days	0.224*	0.272*						
Mean K6 score of students	−0.140	−0.280*						
(f) Healthy School Policies (PO) and Health Related Outcomes								
HKSHQ measures	Primary schools	Secondary Schools						
	PO2.3	PO2.1	PO2.2	PO2.4				
% of students that often obey traffic signals	0.006	0.173	0.260*	0.232				
% of students that often put on seatbelts	−0.124	0.270	0.259*	0.157				
% of students feeling so sad or hopeless that he/she will stop usual activities	−0.226*	−0.079	− 0.025	−0.094				
% of students who are classified as underweight	−0.005	−0.288*	− 0.349**	−0.509**				
Mean Life satisfaction score of students - school experience	−0.022	0.261	0.388**	0.055				
Mean Life satisfaction score of students - overall life	−0.040	0.149	0.296*	−0.151				

The number 1.1 reflect the particular indicator, i.e., School adopts a systematic approach to conduct health education. Can refer to appendix for details of indicators under each key area

**Table 5:**
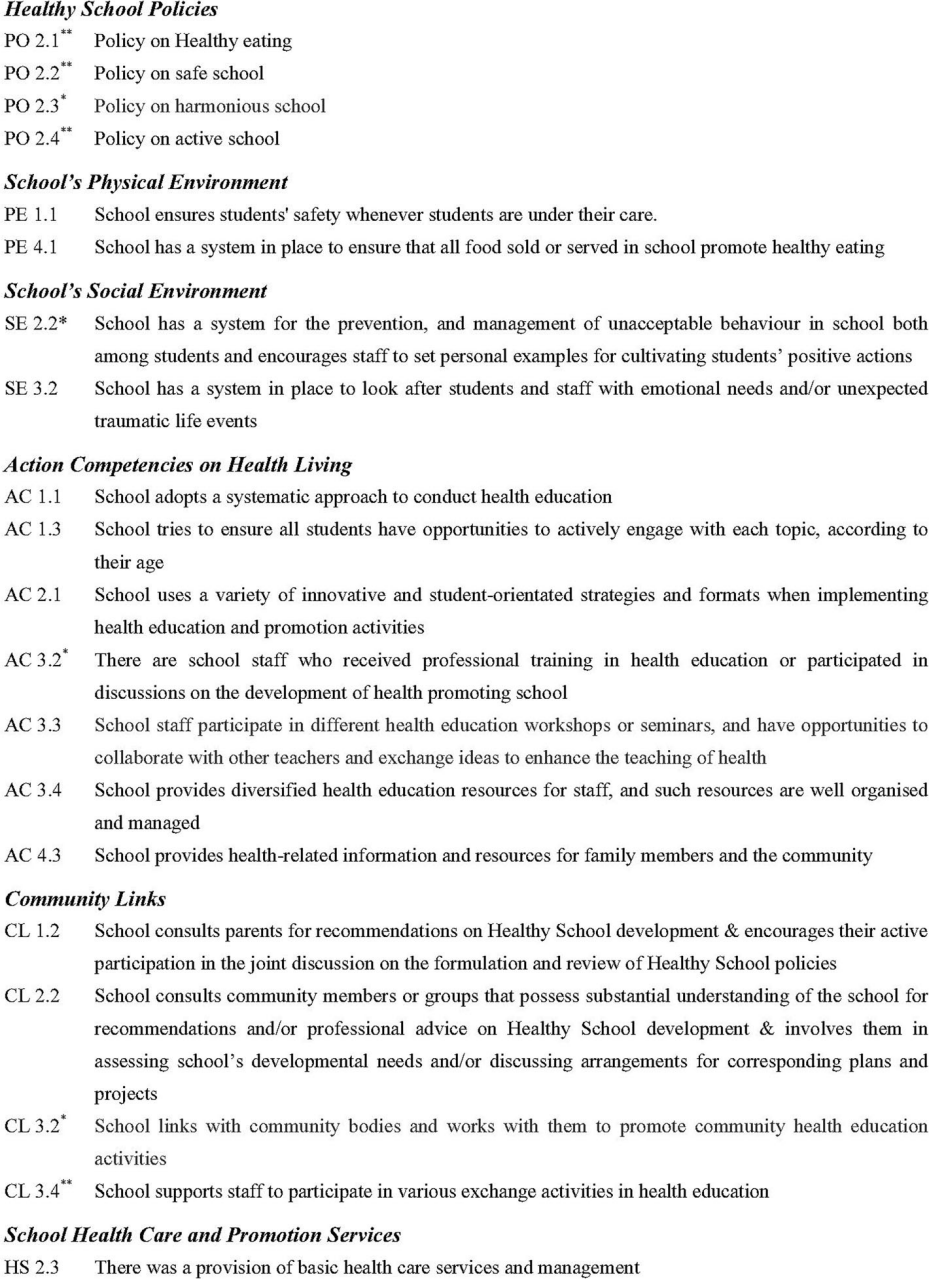
Key indicators for motivating change under each respective key area showing significant impact on health behaviours is listed below(^*^Primary Schools only, **Secondary Schools only)

## Discussion

The core indicators under Action Competencies for Healthy Living (Table 4a) shown to have significant correlation with student health reflect the importance of curriculum design for health education, student engagement, staff professional development and school as resource centre for health information. Literature has highlighted the importance of comprehensive integrated approach via negotiated and systematic planning, leadership and management for effective implementation of HPS [12, 43, 44]. The Austrian study has argued for more systematic and co-ordinated approach with real commitment and understanding among the principals and teachers to integrate health and well-being more deeply into school system rather than many isolated activities notwithstanding enthusiastic support by health promotion service providers [24]. HPS needs to adopt the Critical Health Education (CHE) approach which requires empowerment of students in capacity building developing to act upon the wider social determinants of health [45, 46]. An Ecuadorian study also highlighted the possibility of developing CHE perspective to reaffirm the holistic understanding of health rather than just focusing on biomedical and behavioural modification [46]. Those core indicators reflecting AC analysed by this study (Table 4a) are in line with CHE approach.

Major meta-analysis led by Durlak (2011) examined the effect sizes from 213 school based Social Emotional Learning programs involving 270,034 students from kindergartens to secondary schools. The study found 25% improvement in social and emotional skills, 10% decrease in classroom misbehaviour, anxiety and depression, and 11% improvement in achievement tests in comparison to control sites [47]. More structured health curriculum with boarder coverage and active engagement of students, and enhanced professionalism of teachers in delivering health education including health information for family and community would no doubt enhance better physical and emotional health of the students [48]. Effective school programme in enhancing emotional and social skills of children and adolescents should focus on teaching skills in particular the cognitive, affective and behavioural skills, and competencies [49]. A systematic review has also revealed the positive effects of student participation in school health promotion with regards to increased satisfaction and motivation, positive attitudes, personal development, competencies and knowledge, health related effects, improved interactions and social relationships [48, 50].

With regard to community link, the core indicators reflecting school in consultation with community bodies and parents, and working with them to promote community health education activities, and/or discussion on healthy school policies, have shown significant correlation with self-reported good health and better health as well as better life satisfaction (Table 4b). As school consults community members or professional groups, this would substantiate their understanding of HPS and offer advice and/or support for the holistic approach. Inter-sectoral collaboration would enable the education stakeholders to understand more evidence-based school health promotion, and linking to teaching and learning, and the health stakeholders would also be more upfront with school curriculum development and put health on school agenda with better perception of health status [51]. The same individual, family, school, and community factors predict both positive (e.g., success in schools) and negative (e.g., delinquency) outcomes in school [52]. Therefore, consultation with community and family on health promotion would lead to better health related outcomes.

For school social environment, school having a system in place to look after students and staff with emotional needs and/or unexpected traumatic life events (SE 3.2) has shown significant correlation with better mental health for both primary and secondary school students (Table 4c). SE 2.2 (school has a system for the prevention, and management of unacceptable behaviour in school among students by school teachers responsible for guidance counselling and/or school social workers, and encourages staff to set personal examples for cultivating students’ positive actions) has also been found to correlate significantly with self-reported academic performance, physical active and better mental health (Table 4c). Interventions aiming at reduction of problem behaviours have been shown to enhance emotional and social development of children and adolescents [49]. Preventive work for early identification of students with difficulties and ensuring all parts of the school organization working coherently has found to be effective in promoting emotional and mental health in school [53].

Adolescents will need a supportive school physical environment enabling them to make appropriate food choices promoting health and well-being [8, 27]. Nutrition knowledge transmitted through classroom teaching alone might not be sufficient to influence adolescents’ eating patterns as they need access to healthy food with social support [54]. Therefore, element PE 4.1 (school has a system in place to ensure that all food sold or served in school promote healthy eating) has shown to improve healthy eating in both primary and secondary schools and also physical active in primary school (Table 4d). If school can ensure students’ safety whenever students are under their care (PE 1.1), it has shown to have significant correlation with better mental health and life satisfaction for both primary and secondary schools, and also more physical active in primary schools (Table 4d). Supportive school physical environment is important for health and well-being.

Provision of basic health care services and management (HS 2.3) has shown significant correlation with better self-reported health in both primary and secondary schools and also better mental health for primary schools (Table 4e). Schools would follow the relevant guidelines to establish school health services by their respective authorities. The Austrian study has highlighted the importance of whole school approach with commitment by key stakeholders within the school not just gaining support from health promotion service providers [24]. It is more important to build up a comprehensive, integrative and co-ordinated school health programme [21].

Healthy school policies have shown significant correlation with different aspects of student health (Table 4f). Paper by Gostin et al. has provided evidence that individual will smoke less and eat healthier diets in cities with better regulation [55]. The paper also proposes that law needs not to be coercive but creating environment to make healthy choice easy choice [55]. Findings of the Harvard School of Public Health College Alcohol Study have shown that underage drinking behaviour of the college student could be reduced by additional policy efforts [56]. School policies on health can serve this regulatory role. The School Food Plan in UK with provision of practical steps to improve the quality and take-up of its food in school setting has shown to lead to healthier children as well as improved attainment [57].

HPS model utilises a socio-ecological approach for school-based intervention [33] but schools have found difficulty to implement HPS in entirety [24]. International Union for Health Promotion and Education (IUHPE) produced a document for monitoring and assessing HPS, and recommended schools to use accreditation to measure and track changes [56]. The IUHPE report referenced the HKHSA but did not include evidence whether accreditation type of programme could be effective in motivating changes. Similarly, the design of School Health Index (SHI) by US CDC [58], evaluation framework of National Healthy School Programmes (NHSP) in England [59] and “What is Healthy Together Victoria” Programme in Australia [60] share the auditing type of process in developing action teams, identifying areas of action and progress monitoring. However, they resemble continuous quality improvement but might not inspire change process. Sufficient information is needed to tell why programmes work in some schools and not others [61]. Realistic evaluation using mixed method incorporating both qualitative and quantitative methods involving systematic collection and analysis of data, iterative theory development, strong reflection processes should be adopted [62]. The data collected should also reflect capacity building for schools to understand and making use of the data to integrate health promotion within school setting [63]. The data collection process of HKHSA [14, 23, 34, 39] and further analysis conducted by this study has generated a common set of indicators that would assist evaluation of impact of HPS as discussed in recent paper on HPS [23].

### Limitations

There are limitations in this study. This study only includes school joining the QTN project and those schools would be more committed to implement HPS. The data were collected from the schools participated in HKHSA and they underwent the assessments on voluntary basis. There is always argument that interventions other than HPS would improve health. However, the aim of this study is to identify indicators for success, and it is useful to investigate those schools with diversity of their performance on HPS when they first joined in. There have already been number of studies locally and overseas showing the effectiveness of HPS [9, 13–16, 27, 31, 37, 39, 40]. One should focus on which aspects of HPS are critical for health improvement. Student-centred approach in health education teaching and learning has shown correlation with better health. Therefore, one should explore further how student involvement in other key areas of HPS would have impact on student health. Multivariate linear regression can also be conducted using data to identify correlation between students’ health profile and indicators from the school health profiles, and sociodemographic variables.

This analysis is only cross-sectional. It would be interesting to observe how the school health profile correlates with student health profile in longitudinal study. If longitudinal study can be performed, longitudinal correlation between students’ health related outcomes, and a number of independent variables (indicators from the school health profiles that are significant from the linear regression, location, school, and other socio-demographic variables) can be analysed using Generalized Estimating Equations (GEE).

## Conclusion

There is wide debate what type of data would assist to evaluate impact of HPS, and whether schools should monitor health behaviour outcomes or relying more on process outcomes such as school policies and school environment. For wider implementation of HPS in its entirety, a common set of indicators is needed to identify issues requiring attention that would motivate changes within the school environment as well as health status and behaviours of students. This study has identified the indicators with most significant impact on wide range of health-related outcomes which would serve as key indicators for motivating change. Those indicators could serve as actions for implementation of change at organisational levels for the health and well-being of students. They could also be used as school performance indicators to help schools in monitoring and evaluation of their HPS efforts.

## Additional files

Additional file 1: **Appendix 1**. Health Promoting School Performance Indicator. (PDF 178 kb)

Additional file 2: **Appendix 2**. Additional Basic Requirements for HPS identified by ANOVA. (PDF 213 kb)

Additional file 3: **Appendix 3**.Pearson correlation coefficients between HKSHQ measures and core indicators. (PDF 233 kb)

## Data Availability

Written request could be sent to the corresponding author.

## Abbreviations

AC: Action Competency for Healthy Living
Award: Healthy School Award
CDC: US Center for Disease Control and Prevention
CHE: Critical Health Education
CHEP: Centre for Health Education and Health Promotion
CL: Community Links
HKHSA: Hong Kong Healthy School Awards Scheme
HKSHQ: Hong Kong Health Survey Questionnaire
HPS: Health Promoting School
HS: School Health Care and Promotion Services
IUHPE: International Union for Health Promotion and Education
NCD: Non-communicable Diseases
NHSP: National Healthy School Programmes
PE: School’s Physical Environment
PO: Healthy School Policies
QTN Healthy School: Quality Education Fund Thematic Network of Healthy School
RCT: Randomised Controlled Trial
SDG: Sustainable Development Goal
SE: School’s Social Environment
SHI: Student Health Index
WHO: World Health Organisation
WHSA: The English Wessex Healthy School Award Scheme

## References

[1] United Nations General Assembly. Political declaration of the high-level meeting of the general assembly on the prevention and control of non-communicable diseases. New York: United Nations, 2011. http://www.un.org/ga/search/view_doc.asp?symbol=A/66/L.1&referer=http://www.un.org/en/ga/ncdmeeting2011/&Lang=E. Assessed 10 Aug 2019.

[2] GBD 2013 Risk Factors Collaborators (2015). Global, regional, and national comparative risk assessment of 79 behavioural, environmental and occupational, and metabolic risks or clusters of risks in 188 countries, 1990–2013: a systematic analysis for the Global Burden of Disease Study 2013. Lancet.

[3] Kann L, McManus T, Harris WA, Shanklin SL, Flint KH, Queen B, Lowry R, Chyen D, Whittle L, Thornton J, Lim C, Bradford D, Yamakawa Y, Leon M, Brener N, Ethier KA (2018). Youth Risk Behavior Surveillance - United States, 2017. MMWR Surveill Summ.

[4] Rowe L. This summer, start a small social revolution. Aus Family Physician. 2005;34(1–2):11–2. https://www.racgp.org.au/afpbackissues/2005/200501/200501rowe.pdf. Accessed 10 Aug 2019. 15727350

[5] Lee A, Keung V. Epidemics of childhood obesity amongst Chinese students and effectiveness of school based intervention. Health Education Monograph Series. 2012;29(1):37–46.

[6] WHO. Health Promoting School Framework for Action http://www.wpro.who.int/health_promotion/documents/docs/HPS_framework_for_action.pdf?ua=1. Manila: WHO/WPRO, 2009. Accessed 10 Aug 2019 updated from previous document [WHO Regional Office for the Western Pacific. Health-Promoting Schools Series 5: Regional guidelines. Development of health-promoting schools-A framework for action. Manila: WHO/WPRO, 1996.

[7] Nutbeam D, Clarkson J, Phillips K, Everett V, Hill A, Catford J (1987). The health-promoting school: organisation and policy development in welsh secondary schools. Health Educ J.

[8] Langford R, Campbell R, Magnus D, Bonell CP, Murphy SM, Waters E, Komro KA, Gibbs LF (2014). The WHO health promoting school framework for improving the health and well-being of students and their academic achievement. Cochrane Database Sys Rev.

[9] Lister-Sharp D, Chapman S, Stewart-Brown S, Sowden A (1999). Health promoting schools and health promotion in schools: two systematic reviews. Health Technol Assess.

[10] Inchley J, Muldoon J, Currie C (2006). Becoming a health promoting school: evaluating the process of effective implementation in Scotland. Health Promot Int.

[11] Rowling L, Jeffreys V (2006). Capturing complexity: integrating health and education research to inform health-promoting schools policy and practice. Health Educ Res.

[12] Lee A, Cheung RMB (2017). School as setting to create a healthy teaching and learning environment: using the health promoting school model to foster school-health partnership. J Professional Capital Community.

[13] Moon AM, Mullee MA, Rogers L, Thompson RL, Speller V, Roderick Moon AM, Mullee MA, Thompson RL, Speller V, Roderick P (1999). Health-related research and evaluation in schools. Health Educ.

[14] Lee A, Cheng F, St Leger L (2005). Evaluating health promoting schools in Hong Kong: the development of a framework. Health Promot Int.

[15] Lee A, Cheng FF, Fung Y, St Leger L (2006). Can health promoting schools contribute to the better health and well-being of young people? Hong Kong experience. J Epidemiol Community Health.

[16] Helping schools to become health-promoting environments – an evaluation of the Wessex healthy schools award. Health Promot Int. 1999;14(2):111–22.

[17] Patton GC, Viner RM, Linh LC, Ameratunga S, Fatusi AO, Ferguson BJ, Patel V (2010). Mapping a global agenda for adolescent health. J Adolesc Health.

[18] GBD 2016 SDG Collaborators (2017). Measuring progress and projecting attainment on the basis of past trends of the health-related Sustainable Development Goals in 188 countries: an analysis from the Global Burden of Disease Study 2016. Lancet.

[19] Dooris M (2009). Holistic and sustainable health improvement: the contribution of the settings-based approach to health promotion. Perspectives Public Health.

[20] Dooris M (2013). Expert voices for change: bridging the silos—towards healthy and sustainable settings for the 21^st^ century. Health Place.

[21] Lee, Albert. “School Health Programs in the Pacific Region.” In Oxford Bibliographies in Public Health. Ed. David McQueen. New York: Oxford University Press, 2018. 10.1093/OBO/9780199756797-0173https://www.oxfordbibliographies.com/view/document/obo-9780199756797/obo-9780199756797-0173.xml Accessed 10 Aug 2019.

[22] Rogers E, Moon AM, Mullee MA, Speller VM, Roderick PJ (1998). Developing the ‘health-promoting school’ – a national survey of healthy school awards. Public Health.

[23] Lee A, Keung VM, Lo AS, Kwong AC, Armstrong ES (2014). Framework for evaluating efficacy in health promoting schools. Health Educ.

[24] Adamowitch M, Gugglberger L, Dür W (2017). Implementation practices in school health promotion: findings from an Austrian multiple-case study. Health Promot Int.

[25] Joyce A, Dabrowski A, Aston R, Carey G (2017). Evaluating for impact: what type of data can assist a health promoting school approach?. Health Promot Int.

[26] Nutbeam D (1998). Evaluating health promotion—progress, problems and solutions. Health Promot Int.

[27] St Leger L, Kobe LJ, Lee A, McCall DS, Young IM. School health promotion: achievements, challenges and priorities. In: McQueen D, Jones C (eds). Global Perspective on Health Promotion Effectiveness. New York: Springer, 2007, 107-124. https://link.springer.com/chapter/10.1007/978-0-387-70974-1_8 Accessed 10 Aug 2019.

[28] Lee Albert (2002). Helping schools to promote healthy educational environments as new initiatives for school based management: the Hong Kong Healthy Schools Award Scheme. Promotion & Education.

[29] Lee A, Cheng F, St Leger L (2007). Hong Kong healthy schools team. The status of health promoting schools in Hong Kong and implications for further development. Health Promot Int.

[30] WHO-Regional Office for Europe, European Commission & Council of Europe. First workshop on practice of evaluation of the Health Promoting School: models, experiences and perspectives: executive summary. Copenhagen : WHO Regional Office for Europe, 1999. Held in Switzerland, Bern/Thun: ENHPS 1998. https://apps.who.int/iris/handle/10665/108542 Accessed 10 Aug 2019. Piette D, Roberts C, Prévost M, Tudor-Smith C, Bardolet JT Tracking down ENHPS successes for sustainable development and dissemination: the EVA2 project, Final Report 2002; 24 http://www.euro.who.int/en/health-topics/Life-stages/child-and-adolescent-health/publications/Pre-2005/tracking-down-enhps-successes-for-sustainable-development-and-dissemination-the-eva2-project-final-report. Accessed 10 Aug 2019.

[31] St Leger L (2000). Developing indicators to enhance school health. Health Educ Res.

[32] St Leger L, Young IM (2009). Creating the document ‘promoting health in schools: from evidence to action’. Glob Health Promot.

[33] Chen Fu-Li, Lee Albert (2016). Health-promoting educational settings in Taiwan: development and evaluation of the Health-Promoting School Accreditation System. Global Health Promotion.

[34] Lee A, Cheng FF, Yuen H, Ho M, Lo A, Fung Y, Leung T (2007). Achieving good standard of health promoting schools: preliminary analysis after one year implementation of Hong Kong healthy schools award scheme. Public Health.

[35] CHEP. Student health survey. Centre for Health Education and Health Promotion, The Chinese University of Hong Kong, 2011.

[36] Centers for Disease Control and Prevention. Youth Risk Behaviour Survey. US Department of Health and Human Services, 1999. ftp://ftp.cdc.gov/pub/data/yrbs/1999/YRBS_1999_National_User_Guide.pdf. Accessed 10 Aug 2019.

[37] Kann L, Kinchen SA, Williams BI, Ross JG, Lowry R, Grunbaum JA, Kolbe LJ (2000). Youth risk behaviour surveillance - United States, 1999. J Sch Health.

[38] Lee A, Tsang KK (2004). Youth risk behaviour in a Chinese population: a territory wide youth risk Behavioural surveillance in Hong Kong. Public Health.

[39] Lee A, St Leger L, Moon AS (2005). Evaluating health promotion in schools meeting the needs for education and health professionals: a case study of developing appropriate indictors and data collection methods in Hong Kong. Promot Educ.

[40] Lee A, Keung V, Lo A, Kwong A. Healthy School environment to tackle youth mental health crisis. Hong Kong J Paediatric. 2016;21(2):134–5 http://www.hkjpaed.org/details.asp?id=1064&show=1234 Accessed 10 Aug 2019.

[41] Kessler RC, Green JG, Gruber MJ, Sampson NA, Bromet E, Cuitan M, Furukawa TA, Gureje O, Hinkov H, Hu CY, Lara C (2010). Screening for serious mental illness in the general population with the K6 screening scale: results from the WHO world mental health (WMH) survey initiative. Int J Methods Psychiatr Res.

[42] Huebner ES, Seligson JL, Valois RF, Suldo SM (2006). A review of the brief multidimensional students’ life satisfaction scale. Soc Indic Res.

[43] Samdal O, Rowling L (2011). Theoretical and empirical base for implementation for health-promoting schools. Health Educ.

[44] Simovska V, McNamara PM (eds). School for Health and Sustainability: Theory, Research and Practice. Dordrecht: Springer, 2015. https://www.springer.com/gp/book/9789401791700 Accessed 10 Aug 2019.

[45] Fitzpatrick KJ. Critical approaches to health education. In: Fitzpatrick K, Tinning R, editors. Health Education: Critical Perspectives. New York: Routledge; 2015. https://philpapers.org/rec/FITHEC Accessed 10 Aug 2019.

[46] Torres I. Policy windows for school-based health education about nutrition in Ecuador. Health Promot Int 2017;32(2):331–339. 10.1093/heapro/daw037 Accessed 9 July 2019. 10.1093/heapro/daw037 27169412

[47] Durlak JA, Weissberg RP, Dymnicki AB, Taylor RD, Schellinger KB (2011). The impact of enhancing students’ social and emotional learning: a meta-analysis of school-based universal interventions. Child Dev.

[48] Griebler U, Rojatz D, Simovska V, Forster R (2014). Effects of student participation in school health promotion: a systematic review. Health Promot Int.

[49] Clarke AM, Morreale S, Field CA, Hussein Y, Barry MM. What works in enhancing social and emotional skills development during childhood and adolescence? A review of the evidence on the effectiveness of school-based and out-of-school programmes in the UK. Ireland: World Health Organization Collaborating Centre for Health Promotion Research, National University of Ireland, Galway; 2015. https://aran.library.nuigalway.ie/handle/10379/4981 Accessed 10 Aug 2019.

[50] Catalano RF, Berglund ML, Ryan JA, Lonczak HS, Hawkins JD (2004). Positive youth development in the United States: research findings on evaluations of positive youth development programs. ANNALS Am Acad Political Soc Sci.

[51] Tooher R, Collins J, Braunack-Mayer A, Burgess T, Skinner SR, O'keefe M, Watson M, Marshall HS. Intersectoral collaboration to implement school-based health programmes: Australian perspectives. Health Promot Int 2017;32(2):312–321. 10.1093/heapro/dav120 Accessed 9 July 2019. 10.1093/heapro/dav120 26822033

[52] Weare K. What works in promoting social and emotional wellbeing and responding to mental health problems in schools. London: National Children’s Bureau; 2015. https://www.mentalhealth.org.nz/assets/ResourceFinder/What-works-in-promoting-social-and-emotional-wellbeing-in-schools-2015.pdf. Accessed 10 Auf 2019.

[53] Contento IR. Nutrition education: linking research, theory, and practice. Asia Pac J Clin Nutr. 2008;17(1):176–9 https://www.ncbi.nlm.nih.gov/pubmed/18296331. Accessed 10 Aug 2019. 18296331

[54] Gostin LO, Abou-Taleb H, Roache SA, Alwan A (2017). Legal priorities for prevention of non-communicable diseases: innovations from WHO’s eastern Mediterranean region. Public Health.

[55] Wechsler H, Lee JE, Nelson TF, Kuo M (2002). Underage college students' drinking behavior, access to alcohol, and the influence of deterrence policies: findings from the Harvard School of Public Health College alcohol study. J Am Coll Heal.

[56] Young I, St Leger L, Blanchard C. Monitoring and assessing progress in health promoting schools: issues for policy makers to consider. Saint-Denis, France: International Union for Health Promotion and Education. 2012. https://www.iuhpe.org/index.php/en/iuhpe-thematic-resources/298-on-school-health. Accessed 10 Aug 2019.

[57] Dimbleby H and Vincent J. The School Food Plan. Department of Education, UK, July 2013. https://www.schoolfoodplan.com/wp-content/uploads/2013/07/School_Food_Plan_2013.pdf. Accessed 10 Aug 2019.

[58] Centers for Disease Control and Prevention. School Health Index: a self-assessment and planning guide. CDC, 2014. https://www.cdc.gov/healthyschools/shi/pdf/elementary-total-2014.pdf. Assessed 10 Aug 2019.

[59] Arthur S, Barnard M, Day N, Ferguson C, Gilby N, Hussey D, Morrell G, Purdon S. Evaluation of National Healthy Schools Programme: final report for Department of Health. London: Nat Cen Social Research; 2011. http://natcen.ac.uk/our-research/research/evaluation-of-the-national-healthy-schools-programme/. Accessed 10 Aug 2019.

[60] Department of Health. What is Healthy Together Victoria. https://www2.health.vic.gov.au/about/publications/policiesandguidelines/What-is-Healthy-Together-Victoria. Accessed 10 Aug 2019.

[61] Gleddie D (2012). A journey into school health promotion: district implementation of the health promoting schools approach. Health Promot Int.

[62] Wong G, Greenhalgh T, Westhorp G, Pawson R (2012). Realistic methods in medical education research: what are they and what can they contribute?. Med Educ.

[63] Flaspohler Paul D., Meehan Cricket, Maras Melissa A., Keller Kathryn E. (2012). Ready, Willing, and Able: Developing a Support System to Promote Implementation of School-Based Prevention Programs. American Journal of Community Psychology.

