# Supplementary material for: Effective health promoting school for better health of children and adolescents: indicators for success
Source: BMC Public Health. 2019 Aug 13;19:1088. doi: 10.1186/s12889-019-7425-6 (PMC6691553; doi:10.1186/s12889-019-7425-6)
Supplement: Supplementary file 1 — Appendix 1. Health Promoting School Performance Indicator. (PDF 178 kb) [file 12889_2019_7425_MOESM1_ESM.pdf]

Appendix 1. Health Promoting School Performance Indicator

I Healthy School Policies (PO)

Components	Elements/Indicators
**1. The position of health education and health promotion in school**	1.1. School formulates a set of comprehensive school health policies based on their own needs<br>1.2. A working group or committee was set up to plan, implement and monitor the school health promotion.<br>1.3. Health promotion was included in the school working plan and accorded a high priority when allocating resources for the school year.<br>1.4. Related personnel were consulted in the drawing up, implementing and monitoring the school health policy.
**2. The existence and review of health policies with related personnel being informed and consulted through effective channels**	2.1. Healthy eating.<br>2.2. Safe school.<br>2.3. Harmonious school.<br>2.4. Active school.<br>2.5. Environment-friendly school.<br>2.6. Infectious diseases control.<br>2.7. First aid management.<br>2.8. Contingency for emergency and disasters.<br>2.9. Student healthcare.<br>2.10. Facilitating students on safe use of medicines.<br>2.11. Occupational safety and health.<br>2.12. Prevention of dependent or indulgent behaviour

II School's Physical Environment (PE)

Components	Elements/Indicators
**1. Provision of a safe Environment**	1.1 School ensures students' safety whenever students are under their care.<br>1.2 School has a safe design and organize regular checking and maintenance for the school's building, facilities, amenities and equipments.<br>1.3 Safety is ensured when purchasing new equipment and setting up new facilities; and appropriate precautionary measures are taken.<br>1.4 School has a system in place for the management of emergencies and natural disasters and ensure that all relevant personnel being informed.<br>1.5 School ensures fire safety.<br>1.6 School ensures transportation safety.<br>1.7 School provides a safe and healthy workplace for staff.
**2. Promotion of a hygiene environment**	2.1 School provides a system to develop and sustain a hygienic environment.<br>2.2 School provides an environment to promote and ensure students and staffs' personal hygiene practices.<br>2.3 School encourages students to take good care of school facilities and natural environment.<br>2.4 School provides clean and hygienic washrooms with sufficient sanitary facilities for students and staff.
**3. Provision of a suitable physical environment to enhance learning**	3.1 School provides an adequate and well-maintained ventilation system.<br>3.2 There is sufficient and proper lighting at school.<br>3.3 There is proper sound arrester at school to avoid noisy disturbance from the surrounding areas as much as possible.<br>3.4 School adopts necessary measures to prevent ergonomic problems.<br>3.5 School provides sufficient amenities for students or staffs to rest and relax.
**4. Provision of a healthy eating environment**	4.1 School has a system in place to ensure that all food sold or served in school promote healthy eating.<br>4.2 School provides adequate and hygienic places for students and staff to eat in.<br>4.3 School provides adequate and clean water for use and for drinking.
**5. Promotion of an environment-friendly school**	5.1 School adopts the principles of 'reduce', 'reuse', 'replace' and 'recycle' to promote environment-friendly living and minimise resource consumption.

III School's Social Environment (SE)

Components	Elements/Indicators
**1. Addressing the needs of students and staff**	1.1 School promotes the students' self-confidence and appraise the staff performance through positive reinforcement.<br>1.2 School prohibits inappropriate and non-educational disciplinary education punishment.<br>1.3 School involves students in policy making (NB: Not applicable to special school)<br>1.4 School promotes the development of students' skills in leadership, communication and inter-personal relationship.
**2. Creating an environment of friendliness and care in school**	2.1 School sets up a system to provide mutual support and care for the students and staff.<br>2.2 School has a system for the prevention, and management of unacceptable behaviour in school both among students and encourages staff to set personal examples for cultivating students' positive actions.
**3. Assistance for students and staff with special needs**	3.1 School has a system in place and carry out appropriate actions to facilitate students with special needs.<br>3.2 School has a system in place to look after students and staff with emotional needs and/or unexpected traumatic life events.
**4. Establishment of an inclusive environment of value and mutual respect**	4.1 School has a system in place to ensure equal opportunities among students and staff and provides channels to let the comments and recommendations made by both parties be expressed and heard..<br>4.2 School encourages students and staff to respect and value each other's individuality and differences amongst cultures, genders, religions, disadvantaged groups and races.

IV Action Competencies for Healthy Living (AC)

The school offers health education to students according to their age and educational level in the forms of curriculum and associated activities where students gain knowledge and healthy life skills related to the **Ten Health Content Areas**. The areas include: Personal Health; Food and Nutrition; Mental and Emotional Health; Family Life and Sex Education; Prevention and Management of Disease; Smoking, Alcoholism, Drug Use and Abuse; Consumer Health; Safety and First Aid; Environmental Health and Conservation; Life, Ageing and Death.

Components	Elements/Indicators
**1. Comprehensive curriculum with health related issues for students to acquire health skills**	1.1. School adopts a systematic approach to conduct health education.<br>1.2. School provides time for students to explore and comprehend each Content Area.<br>1.3. School tries to ensure all students have opportunities to actively engage with each topic, according to their age.<br>1.4. School uses various methods to evaluate the learning outcome of students and teaching effectiveness.
**2. Strategic approaches for students to acquire health skills**	2.1 School uses a variety of innovative and student-orientated strategies and formats when implementing health education and promotion activities.<br>2.2 School provides students from all grades with sufficient physical education lessons per week and a wide range of co-curricular activities that promote physical activities for all students.<br>2.3 School creates suitable learning and teaching environment to stimulate and facilitate learning process.
**3. Staff are well-equipped to promote health**	3.1 School develops policy on staff health education training. Appropriate measures are in place to encourage and facilitate staff to receive the training.<br>3.2 There are school staff who received professional training in health education or participated in discussions on the development of health promoting school.<br>3.3 School staff participate in different health education workshops or seminars, and have opportunities to collaborate with other teachers and exchange ideas to enhance the teaching of health.<br>3.4 School provides diversified health education resources for staff, and such resources are well organised and managed.
**4. Related health skills for family members and the community**	4.1 School actively organises health education and health-promoting activities for family members.<br>4.2 School actively organises health education and health-promoting activities for the community.<br>4.3 School provides health-related information and resources for family members and the community.

V Community Links (CL)

Components	Elements/Indicators
**1. Family involvement in school affairs**	1.1 School forms a parent-teacher association and acts as a bridge between parents and the school to collaborate and discuss school and health-related affairs<br>1.2 School consults parents for recommendations on Healthy School development & encourages their active participation in the joint discussion on the formulation and review of Healthy School policies.<br>1.3 School provides opportunities for parents to work closely with the school, including the organisation of, assistance and participation in school and health-related activities.<br>1.4 School actively organises health-promoting activities for parents and their family members. (NB: Aspects of physical, psychological, social and spiritual health can be considered, including parent education)<br>1.5 School establishes a system of close and mutual communication is in place with parents.
**2. Community involvement in school development**	2.1 School actively encourages community members or groups to participate in the joint discussion on the formulation and review of Health School policies.<br>2.2 School consults community members or groups that possess substantial understanding of the school for recommendations and/or professional advice on Healthy School development & involves them in assessing school's developmental needs and/or discussing arrangements for corresponding plans and projects<br>2.3 School collaborates with government departments, local agencies and community groups to organise health-promoting activities.
**3. Proactive linkage with other community bodies**	3.1 School establishes networks with schools and works with them to promote school health promotion.<br>3.2 School links with community bodies and works with them to promote community health education activities.<br>3.3 School supports students to participate in various community, health-promoting activities and social services.<br>3.4 School supports staff to participate in various exchange activities in health education.<br>3.5 School encourages students to make full use of community resources to promote their personal development.

VI School Health Care and Promotion Services (HS)

Components	Elements/Indicators
**1. Infectious Disease Control**	1.1 School has a system for monitoring, control and management of infectious diseases.<br>1.2 School encourages students to be immunised against appropriate infectious disease and their immunization status should be properly documented and followed up. (NB: Immunization records of ALL students, including those admitted in the middle of the term, were kept in Secondary School.)
**2. Basic Health Care Services for Students**	2.1. School encourages students to have health screening at least once a year with a monitoring system in place.<br>2.2. School encourages students to have regular dental check with a monitoring system in place. (NB: Education on oral health may be provided in Secondary School and school is encouraged to keep track on the uptake rate of dental check.)<br>2.3. There was a provision of basic health care services and management.<br>2.4. School provides basic management and care for students with special needs.
**3. Record of Student Health Status**	3.1 All students' heights and weights was regularly monitored and followed up with their families when needed.<br>3.2 School is aware of spinal health of the students and measures are in place for monitor and provision of health education with appropriate follows up actions, such as monitor the weights of school bags at least annually for Primary school student and provide education on spinal health.<br>3.3 School develops and maintain individual, comprehensive and up-to-date student's health records.
**4. Psychological and Counselling Services**	4.1 There was psychological and counselling services available for students and their parents or guardians
**5. Emergency Services**	5.1 School has at least **TWO** qualified first-aiders.<br>5.2 School has specified procedures regarding first aid arrangement for large-scale activities and special events.<br>5.3 School provides standard first aid box(es) and equipment which must be checked regularly for defects and expiry .<br>5.4 There was emergency management systems in place for the injured.
**6. Occupational Health**	6.1 School actively promotes occupational health and support related training.<br>6.2 School promotes staff health through active encouragement of regular health screening, up to date immunisation, and other health activities. School may also maintain confidential records of all staff health.<br>6.3 School has a system in place to cater for staff's social needs and to support staff with emotional needs.<br>6.4 School provides a safe and hazard free working environment for all staff.
**7. Health Services Information**	7.1 School will provide latest information on health or related services from relevant organizations for the development of health services.
