# Supplementary material for: Effective health promoting school for better health of children and adolescents: indicators for success
Source: BMC Public Health. 2019 Aug 13;19:1088. doi: 10.1186/s12889-019-7425-6 (PMC6691553; doi:10.1186/s12889-019-7425-6)
Supplement: Supplementary file 2 — Appendix 2. Additional Basic Requirements for HPS identified by ANOVA. (PDF 213 kb) [file 12889_2019_7425_MOESM2_ESM.pdf]

Appendix 2. Additional Basic Requirements for HPS identified by ANOVA

	**Mean score of indicator in schools with**			
**Element**	**Gold Award n=16**	**Silver Award n=23**	**Bronze Award n=15**	**Baseline Assessment n=104**
Healthy School Policies (PO)				
2.1 Healthy Eating	0.87	0.80	0.71	0.52
2.2 Safe School	0.82	0.75	0.66	0.51
2.3 Harmonious School	0.89	0.85	0.76	0.59
2.4 Active School	0.85	0.80	0.69	0.33
School's Physical Environment (PE)				
1.1 School ensures students' safety whenever students are under their care	0.82	0.80	0.76	0.72
1.4 School has a system in place for the management of emergencies and natural disasters and ensure that all relevant personnel being informed	0.83	0.81	0.76	0.71
1.5 School ensures fire safety	0.88	0.83	0.81	0.79
1.7 School ensure a safe and healthy workplace for staff	0.64	0.60	0.59	0.52

School's Social Environment (SE)				
2.2 School has a system for the prevention, and management of unacceptable behaviour in school both among students and encourages staff to set personal examples for cultivating students' positive actions	0.81	0.77	0.73	0.65
Action Competencies for Healthy Living (AC)				
1.3 School tries to ensure all students have opportunities to actively engage with each topic, according to their age	0.81	0.73	0.64	0.43
3.2 There are school staff who received professional training in health education or participated discussions on the development of HPS	0.75	0.38	0.31	0.24
3.3 School staff participate in different health education workshops or seminars, and have opportunities to collaborate with other teachers and exchange ideas to enhance the teaching of health	0.84	0.77	0.66	0.52
3.4 School provides diversified health education resources for staff, and such resources are well organised and managed	0.89	0.83	0.79	0.68

Community Links (CL)				
2.2 School consults community members or groups that possess substantial understanding of the school for recommendations and/or professional advice on Healthy School development and involves them in assessing school's developmental needs and/or discussing arrangements for corresponding plans and projects	0.87	0.71	0.63	0.45
School Health Care and Promotion Services (HS)				
2.3 There was a provision of basic health care services and management	0.85	0.80	0.76	0.67

p-value < 0.05
