# Supplementary material for: Effective health promoting school for better health of children and adolescents: indicators for success
Source: BMC Public Health. 2019 Aug 13;19:1088. doi: 10.1186/s12889-019-7425-6 (PMC6691553; doi:10.1186/s12889-019-7425-6)
Supplement: Supplementary file 3 — Appendix 3.Pearson correlation coefficients between HKSHQ measures and core indicators. (PDF 233 kb) [file 12889_2019_7425_MOESM3_ESM.pdf]

Appendix 3. Pearson correlation coefficients between HKSHQ measures and core indicators (** p-value <0.01, * p-value<0.05)

(a) Action Competencies for Healthy Living (AC) and Health Related Outcomes

	**Primary schools**							**Secondary Schools**						
**HKSHQ measures**	**AC1.1**	**AC1.3**	**AC2.1**	**AC3.2**	**AC3.3**	**AC3.4**	**AC4.3**	**AC1.1**	**AC1.3**	**AC2.1**	**AC3.2**	**AC3.3**	**AC3.4**	**AC4.3**
% of students think they are having good academic performance in past 12 months	0.175	0.138	0.085	0.155	0.260*	-0.043	0.023	***0.367*****	0.064	0.121	-0.015	0.206	0.112	0.193
% of students think they are having good health status over past 30 days	***0.314*****	***0.349*****	***0.234****	***0.256****	***0.276*****	-0.003	0.118	0.108	0.016	0.003	-0.083	-0.122	-0.063	-0.064
% of students that often obey traffic signals	***0.258****	0.034	***0.229****	0.075	0.259*	0.003	***0.248****	***0.400*****	***0.269****	***0.308****	-0.027	0.260*	-0.062	0.112
% of students that often put on seatbelts	0.166	0.004	0.126	0.171	0.230*	0.006	0.171	***0.370*****	0.182	***0.275****	-0.038	0.182	0.042	0.239
% of students having enough vegetable every day	0.152	0.186	-0.011	***0.236****	***0.253****	0.036	0.001	-0.055	-0.164	-0.242	-0.272*	-0.174	-0.052	-0.163
% of students having enough fruit every day	***0.243****	***0.303*****	0.099	***0.264****	***0.269****	0.088	0.158	0.258	0.064	0.147	-0.159	0.115	0.345**	0.194
% of students having crisps more than 4 times per week	-0.149	***-0.348*****	-0.069	-0.136	-0.029	-0.156	-0.046	***-0.267****	-0.158	0.058	0.208	-0.047	0.029	-0.010
% of students having candies more than 4 times per week	***-0.266****	***-0.487*****	-0.177	-0.167	-0.179	-0.250*	-0.075	***-0.427*****	-0.138	-0.110	-0.099	-0.103	-0.051	***-0.333****
% of students having soft drink more than 4 times per week	***-0.326*****	***-0.356*****	-0.192	***-0.287*****	-0.166	-0.062	***-0.214****	-0.193	-0.083	-0.158	0.091	0.010	-0.019	-0.058
% of students having preserved meat more than 4 times per week	***-0.211****	***-0.291*****	-0.101	***-0.295*****	-0.131	-0.010	-0.064	***-0.395*****	-0.189	-0.137	-0.054	-0.038	-0.064	-0.129

HKSHQ measures	Primary schools							Secondary Schools						
	**AC1.1**	**AC1.3**	**AC2.1**	**AC3.2**	**AC3.3**	**AC3.4**	**AC4.3**	**AC1.1**	**AC1.3**	**AC2.1**	**AC3.2**	**AC3.3**	**AC3.4**	**AC4.3**
% of students having enough physical activity	***0.330*****	***0.281*****	0.206	***0.240****	***0.280*****	0.030	***0.318*****	0.094	-0.051	-0.032	-0.013	-0.043	-0.045	-0.035
% of students spend 2 hours or more on watching TV every day	***-0.255****	***-0.215****	***-0.303*****	-0.075	***-0.237****	***-0.295*****	***-0.331*****	***-0.463*****	-0.111	-0.220	-0.075	-0.128	-0.199	***-0.415*****
% of students spend 2 hours or more on playing video games every day	-0.028	0.072	-0.095	-0.179	-0.117	-0.189	-0.205	-0.249	-0.070	-0.170	-0.003	-0.185	***-0.328****	***-0.288****
Mean K6 score of students	***-0.323*****	***-0.359*****	-0.205	***-0.362*****	***-0.277*****	0.031	***-0.246****	***-0.372*****	***-0.363*****	***-0.307****	-0.174	-0.036	-0.211	***-0.299****
% of students feeling so sad or hopeless that he/she will stop usual activities	-0.088	-0.034	-0.153	-0.067	-0.078	0.002	***-0.239****	-0.085	-0.082	-0.098	0.228	0.037	-0.028	0.018
% of students smoke	-0.078	0.080	-0.046	***-0.248****	-0.070	-0.007	-0.057	-0.115	-0.142	***-0.267****	0.037	-0.175	0.013	-0.082
% of students have sex	0.110	0.056	0.186	0.000	-0.021	0.102	0.022	***-0.265****	-0.191	***-0.310****	-0.086	-0.260*	0.016	-0.128
% of students who are classified as underweight	0.009	-0.153	-0.021	-0.085	-0.044	0.008	0.064	***-0.305****	***-0.301****	***-0.396*****	-0.138	***-0.310****	-0.086	-0.199
Mean Life satisfaction score of students - family life	0.200	0.125	***0.271****	***0.236****	***0.271****	0.018	***0.299****	0.062	0.045	0.245	0.077	-0.028	0.121	0.060
Mean Life satisfaction score of students - friendship	0.066	-0.035	0.098	0.231	0.202	-0.013	***0.237****	-0.068	0.192	0.231	0.073	0.035	0.115	-0.051
Mean Life satisfaction score of students - school experience	-0.050	-0.153	0.042	0.183	0.008	-0.113	0.078	0.219	0.244	***0.384*****	0.187	0.235	0.137	***0.292****
Mean Life satisfaction score of students - themselves	***0.262****	0.039	***0.348*****	0.061	***0.376*****	-0.005	***0.459*****	0.126	0.167	0.220	0.288*	0.098	0.265	***0.295****

HKSHQ measures	Primary schools							Secondary Schools						
	AC1.1	AC1.3	AC2.1	AC3.2	AC3.3	AC3.4	AC4.3	AC1.1	AC1.3	AC2.1	AC3.2	AC3.3	AC3.4	AC4.3
Mean Life satisfaction score of students - living environment	0.218	0.042	0.225	***0.285****	0.230	-0.006	***0.338*****	0.139	0.063	0.271	0.244	0.138	0.122	0.117
Mean Life satisfaction score of students - overall life	0.155	0.034	0.193	0.186	***0.259****	-0.046	***0.347*****	0.107	0.094	***0.291****	0.270	0.121	0.098	0.073

(b) Community Links (CL) and Health Related Outcomes

HKSHQ measures	Primary schools				Secondary schools			
	CL1.2	CL2.2	CL3.2	CL3.4	CL1.2	CL2.2	CL3.2	CL3.4
% of students think they are having good health status over past 30 days	0.110	***0.370*****	***0.351*****	0.209	-0.016	***0.274****	0.040	0.111
% of students that often obey traffic signals	***0.270****	0.121	0.037	0.138	***0.261****	0.257	0.235	0.273*
% of students that often put on seatbelts	***0.270****	0.118	-0.026	-0.011	***0.284****	0.272*	0.053	0.344**
Mean K6 score of students	-0.058	***-0.375*****	-0.057	-0.278	-0.119	***-0.293****	-0.046	-0.397**
Mean Life satisfaction score of students - family life	***0.268****	***0.328*****	0.177	0.169	-0.047	0.040	0.122	0.208
Mean Life satisfaction score of students - school experience	0.005	0.029	-0.069	-0.008	0.172	0.193	0.061	***0.340****
Mean Life satisfaction score of students - themselves	***0.235****	***0.305*****	0.075	0.030	0.056	0.280	0.011	0.239
Mean Life satisfaction score of students - living environment	0.193	***0.255****	-0.005	0.239*	0.099	0.133	-0.055	0.203
Mean Life satisfaction score of students - overall life	0.155	***0.292****	0.025	0.107	0.081	0.128	0.006	0.240

(c) School's Social Environment (SE) and Health Related Outcomes

HKSHQ measures	Primary schools		Secondary Schools	
	SE2.2	SE3.2	SE2.2	SE3.2
% of students think they are having good academic performance in past 12 months	***0.223****	0.037	0.142	0.174
% of students having enough physical activity	***0.252****	0.190	-0.056	0.006
% of students having K6 score >12	***-0.259****	***-0.241****	-0.157	***-0.300****
Mean K6 score of students	***-0.211****	***-0.291******	-0.241	-0.248

(d) School's Physical Environment (PE) and Health Related Outcomes

HKSHQ measures	Primary Schools					Secondary schools				
	PE1.1	PE1.4	PE1.5	PE1.7	PE4.1	PE1.1	PE1.4	PE1.5	PE1.7	PE4.1
% of students having enough fruit every day	0.119	-0.208	0.217*	0.155	0.164	0.194	0.150	0.082	0.330*	***0.345******
% of students having soft drink more than 4 times per week	-0.278**	0.161	-0.194	-0.142	***-0.282******	-0.046	0.067	-0.026	0.024	0.082
% of students having enough physical activity	***0.216****	-0.061	0.302**	0.068	***0.239****	0.248	-0.048	0.203	0.124	0.055
Mean K6 score of students	***-0.227****	0.269*	-0.277**	-0.160	-0.183	***-0.284****	0.119	-0.295*	-0.173	-0.302*
Mean Life satisfaction score of students - living environment	***0.265****	0.026	0.106	0.110	0.220	***0.335****	0.041	0.401**	0.066	0.072
Mean Life satisfaction score of students - overall life	***0.258****	-0.056	0.181	0.093	0.183	0.196	-0.019	0.351*	-0.020	0.110

(e) School Health Care and Promotion Services (HS) and Health Related Outcomes

HKSHQ measures	Primary Schools				Secondary Schools			
	HS1.2	HS2.1	HS2.3	HS6.1	HS1.2	HS2.1	HS2.3	HS6.1
% of students think they are having good health status over past 30 days	-0.014	0.092	***0.224****	0.149	0.187	0.251	***0.272****	0.188
Mean K6 score of students	0.101	-0.115	-0.140	-0.138	-0.342	-0.249	***-0.280****	-0.446**

(f) Healthy School Policies (HS) and Health Related Outcomes

HKSHQ measures	Primary schools				Secondary Schools			
	PO2.1	PO2.2	PO2.3	PO2.4	PO2.1	PO2.2	PO2.3	PO2.4
% of students that often obey traffic signals	-0.023	-0.052	0.006	0.041	0.173	***0.260****	0.160	0.232
% of students that often put on seatbelts	-0.065	-0.060	-0.124	-0.152	0.270	***0.259****	0.029	0.157
% of students feeling so sad or hopeless that he/she will stop usual activities	-0.087	-0.227*	***-0.226****	-0.066	-0.079	-0.025	-0.009	-0.094
% of students who are classified as underweight	-0.007	0.017	-0.005	-0.084	***-0.288****	-0.349**	-0.247	***-0.509*****
Mean Life satisfaction score of students - school experience	-0.104	-0.075	-0.022	-0.169	0.261	***0.388*****	0.049	0.055
Mean Life satisfaction score of students - overall life	0.055	0.060	-0.040	-0.143	0.149	***0.296****	-0.163	-0.151
